# PD-L1 expression in keratinocyte and infiltration of CD4 + T lymphocyte can predict a severe type of erythema multiforme major induced by the anti-PD-1 antibody, pembrolizumab

**DOI:** 10.1007/s13691-024-00676-4

**Published:** 2024-03-29

**Authors:** Ryohei Kadoi, Taichi Yoshida, Mai Noto, Aya Toyoshima, Sino Fujii, Koji Fukuda, Kazuhiro Shimazu, Daiki Taguchi, Hanae Shinozaki, Naoki Kodama, Michihiro Kono, Hiroshi Nanjyo, Hiroyuki Shibata

**Affiliations:** 1 Department of Clinical Oncology, Graduate School of Medicine, Akita University, Hondo 1-1-1, Akita, Japan; 2 Department of Dermatology and Plastic Surgery, Graduate School of Medicine, Akita University, Akita, Japan; 3 Division of Nurse Practitioner, Akita University Hospital, Akita, Japan; 4 Department of Pathology, Akita University Hospital, Akita, Japan

**Keywords:** Anti-PD-1 antibody, Erythema multiforme major, Severe cutaneous adverse reaction, PD-L1 expression, CD8^+^ T cell

## Abstract

Skin toxicity is the most common adverse event of treatment with immune check point inhibitors. Among them, erythema multiforme is a rare occurrence with a frequency of 4%, with most of the cases developing grade 1/2 disease. We experienced high grade erythema multiforme major developing with pembrolizumab treatment for anal canal cancer with extensive skin metastases. Steroid ointment was ineffective, and the skin lesions with blisters expanded to > 45% of the body surface area. The patient was at risk for symptom aggravation, and a pulse therapy with methylprednisolone and increasing the dose of oral prednisolone (1 mg/kg) were started. The skin lesions improved in 1.8 months. Unless urgent and appropriate treatments such as high dose steroid administration were conducted, the skin toxicities could not be controlled. The presence of CD4^+^ T cells and PD-L1^+^ keratinocytes in the skin biopsy might be a predictive marker of erythema multiforme major resistant to standard steroid treatment.

**Supplementary Information:**

The online version contains supplementary material available at 10.1007/s13691-024-00676-4.

## Introduction

Immune checkpoint inhibitors (ICIs) have dramatically improved the therapeutic outcomes of many cancers [1]. Pembrolizumab (Pem), an anti-PD-1 antibody, was approved for the treatment of unresectable or metastatic solid tumors with mutational burden-high (TMB-H) [≥ 10 mutations/mega-base (mut/Mb)] [2]. The side effects related to ICIs are referred to as immune-related adverse events (irAEs) caused by especially T cells [3]. IrAEs occur in 90% and 70% of patients treated with anti-CTLA-4 and anti-PD-1/PD-L1 respectively [3].

The most frequent skin irAEs include grade 1 and 2 (G1/2) pruritus (24%), G1/2 rashes (21%), G1/2 vitiligo (10%), and G1/2 dry skin [4]. Erythema multiforme (EM) rarely occurs, with G1/2 EM reported in only 4% of cases, and G3/4, EM major, being very rare [4]. Here, we describe a case of EM major after treatment with pembrolizumab resistant to standard steroid treatment.

## Case report

**Fig. 1:**
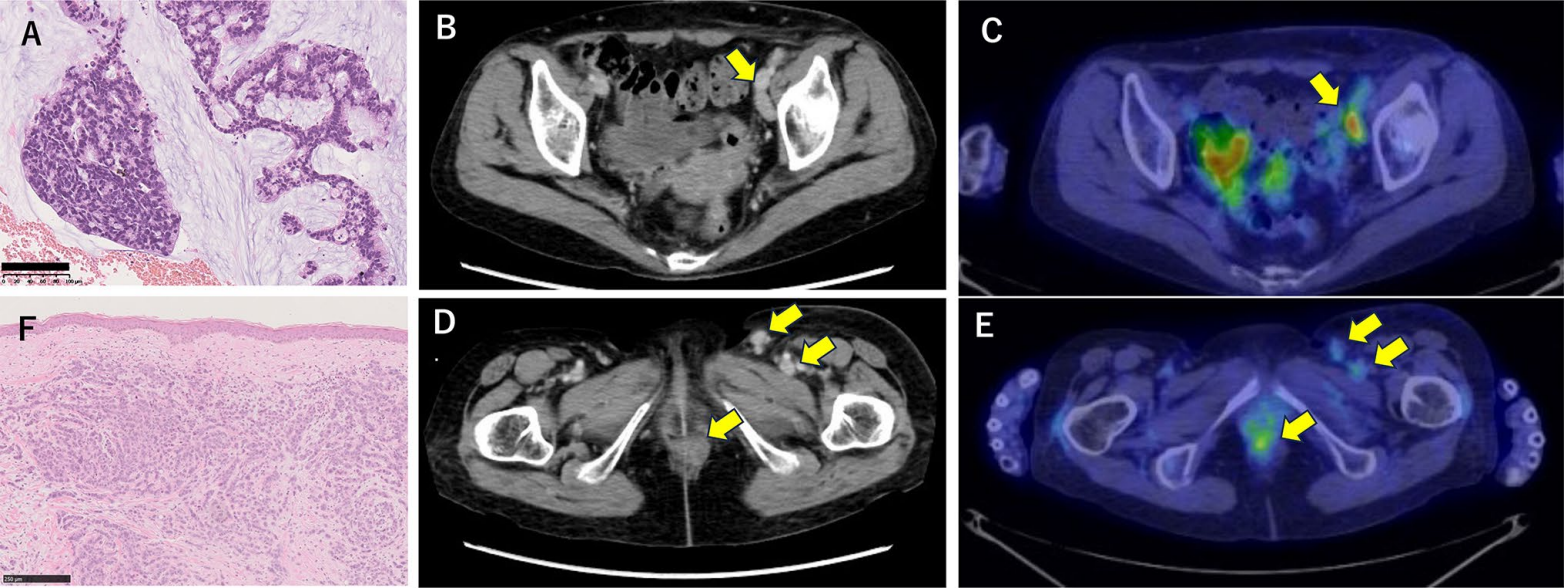
Presentation of anal canal cancer. **A** A biopsy specimen of anal canal cancer (mucinous adenocarcinoma). The black bar indicates 100 μm. **B** Computed tomography (CT) imaging of lymph node metastasis around the external and internal iliac arteries. **C** Positron emission tomography (PET) imaging of (**B**). **D** CT imaging of the anal canal cancer and inguinal lymph node metastasis. **E** PET imaging of CD. **F** Biopsy specimen of abdominal wall metastasis. The black bar indicates 250 μm

A 70-year-old woman palpated a tumor on her perianal area, which was accompanied by pain, prompting a visit to her former doctor in August 2021. Histopathological examination revealed that the tumor was a mucinous adenocarcinoma, leading to diagnosis of an anal canal cancer (Fig. 1A). Computed tomography (CT) and positron emission tomography showed multiple lymph node metastases (Fig. 1B–E). She was then referred to our department to receive chemotherapy in October 2021. Systemic chemotherapies according to the treatment for colorectal cancer (CRC) were conducted sequentially as follows: mFOLFOX6 + cetuximab, capecitabine + bevacizumab, FOLFIRI + panitumumab, and trifluridine/tipiracil + bevacizumab from October 2021 to October 2022. In January 2022, skin lesions were apparent on her left femur and abdominal wall. A skin biopsy indicated that these lesions were metastatic lesions from anal canal cancer (Fig. 1F). Radiation and electron beam irradiation (EBI) were also conducted. Almost all drugs for CRC became ineffective and cancer genome testing (FoundationOne CDx, FOUNDATION MEDICINE, INC. MA, USA) was conducted. TMB-H (11 mut/Mb) was detected. Treatment with Pem was started from December 2022. EBI was conducted for the new skin metastases of the left hip and right lower abdomen in August 2022.

**Fig. 2:**
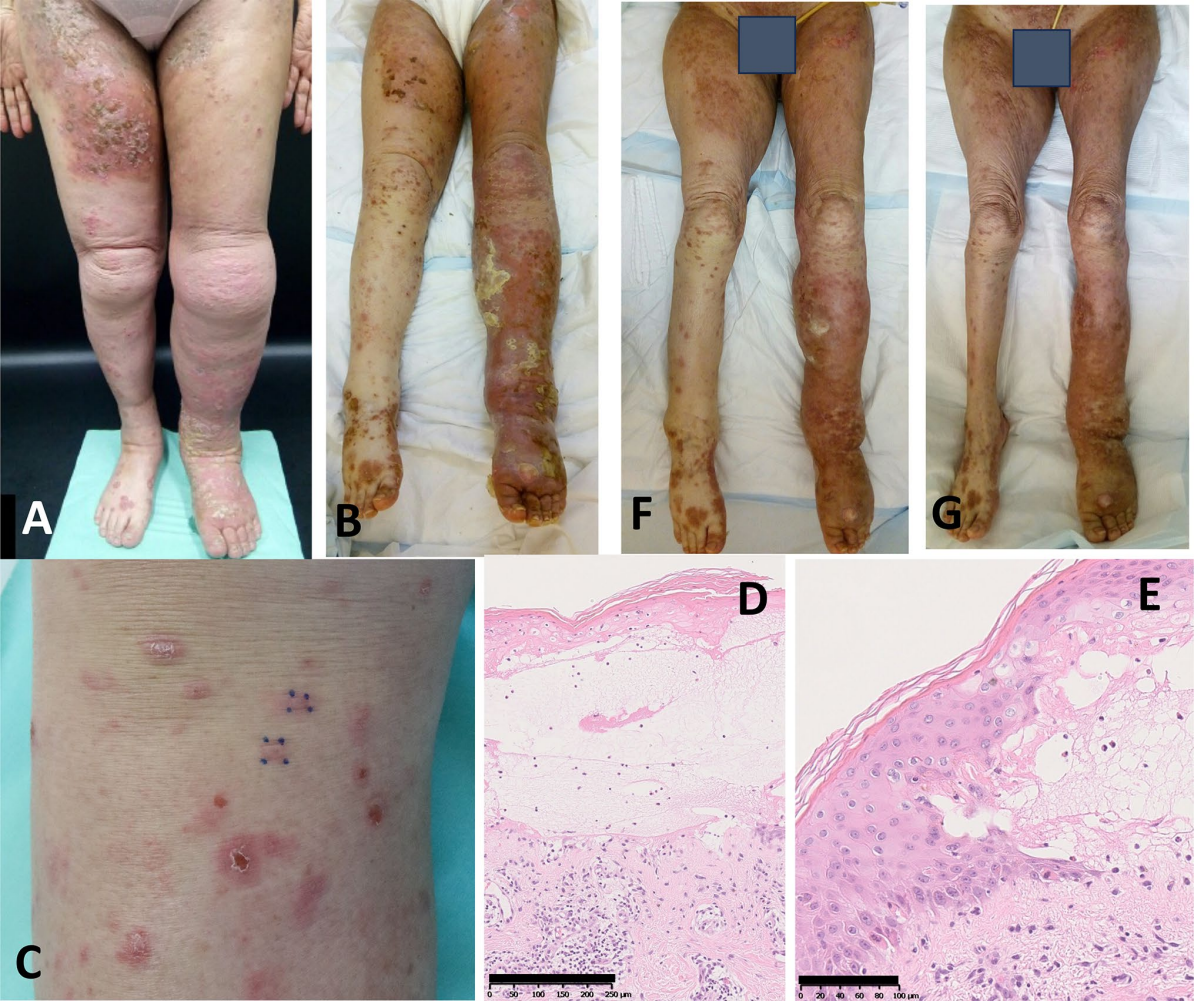
Presentation of skin lesions. **A** Skin lesions on the day of admission (April 2023). **B** Skin lesions on day 17. Lesions were consistent with “Bullous Dermatoses” in ASCO guideline. **C** The edematous erythemata, which are called “Bullous Dermatoses” lesions in ASCO guideline was sampled as biopsy specimen. **D**–**E** The biopsy specimen shows that the subepidermal blister is seen in the center of the lesion (**D**) and that the interface and perivascular dermatitis with sparse eosinophils in dermis and prominent dyskeratotic keratinocytes in epidermis is seen in the margin of the lesion (**E**). **F** Skin lesions on day 45. **G** Skin lesions on day 54. The black bars in (**D**) and (**E**) indicate 250 μm and 100 μm, respectively

In March 2023, EBI was also conducted for the new skin metastases in the lower abdomen, vulva, and right femur. In April 2023, after five courses of Pem, both of her lower extremities were swollen with exudates, many irregular erythemas, blisters, and pustules (Fig. 2A). Also, edematous erythemata and bullae were on her trunk and upper extremities. As she had severe general fatigue and a fever in the 38℃, she had difficulty in walking. She was diagnosed as having a suspicious erythema multiforme; therefore, she was admitted. A topical steroid ointment (0.05% betamethasone butyrate propionate) was started. The laboratory data were as follows: WBC 7200 /µL, Hb 10.8 mg/dL, Plt 554 × 10^3^/µL, TP 5.6 g/dL, Alb 2.7 g/dL, AST/ALT 20/11 U/L, ALP 58 U/L, T-Bil 0.4 mg/dL, LDH 141 IU/L, BUN/Cr 19.5/1.35 mg/dL, Na/K/Cl 135/5.1/97 mEq/L, corrected Ca 10.3 mg/dL, CRP 3.89 mg/dL. The data indicated the presence of a previous infection of Epstein‐Barr virus, herpes simplex virus and varicella zoster virus was detected. A possibility of active viral infection was denied. The anti-bullous pemphigoid 180 antibody (BP180) level was 4.7 U/L (normal limit: < 9.0). Anti-desmoglein 1 and 3 antibodies, which are the markers of pemphigus vulgaris [5], were < 3.0 U/mL (both normal limits: < 20.0 U/mL).

**Fig. 3:**
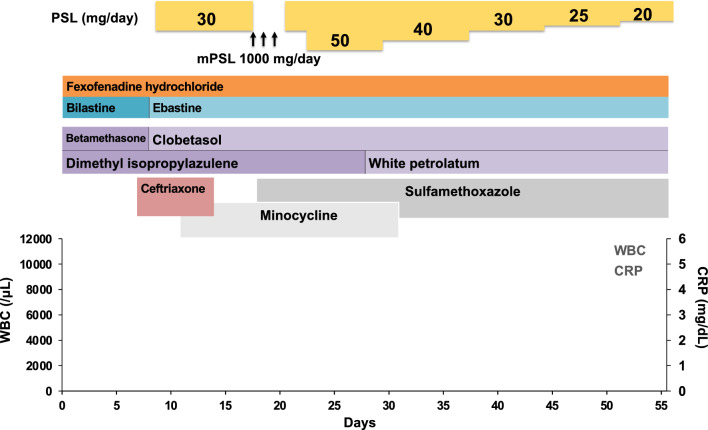
Schematic summary of the treatment protocol. *PSL* prednisolone, *mPSL* methylprednisolone. Antibiotics such as ceftriaxone, minocycline, and sulfamethoxazole, were used promptly. Dimethyl isopropylazulene was used for the inflammation. White petrolatum was used to moisture her skin. Fexofenadine hydrochloride, an antihistamine, bilastine, or ebastine, nonsedating second generation antihistamines were used to relieve allergy symptoms

At day 8, the blisters expanded to the abdominal wall. Her skin lesion was highly suspicious of skin irAE, rash or inflammatory dermatitis (RID) (Grade 2), judging from the skin lesion coverage of 36% of the body surface area (BSA) by rule of nine without mucosal lesions and ASCO guideline [6]. Oral prednisolone (30 mg/day = 0.6 mg/kg body weight) was administered. Further, 0.05% betamethasone butyrate propionate ointment was changed to 0.05% clobetasol propionate ointment. However, her skin toxicities were not improved, and the skin lesion coverage grew up to 45% of BSA (Fig. 2B). The biopsy of the edematous erythemata on her right lower leg (Fig. 2C) indicated a subepidermal blister in center of the lesion (Fig. 2D) and interface and perivascular dermatitis with sparse eosinophils in dermis and prominent dyskeratotic keratinocytes in epidermis in the margin of the lesion (Fig. 2E). Direct immunofluorescence revealed C3 deposits but neither of IgG, IgA or IgM deposit along the dermal–epidermal junction. Thus, those were diagnosed as bullous EM. Furthermore, a genital erosion was observed, which was categorized as RID (Grade 3/4) or bullous dermatoses (BD) (Grade 3/4). This case presents with erythema multiforme with relatively mild mucosal lesions distributed predominantly on the extremities. On the other hand, Stevens–Johnson syndrome (SJS)/ toxic epidermal necrolysis (TEN) presents with widespread mucosal lesions, with erythema predominantly distributed on the face and trunk. Based on the above differential diagnosis, this case was considered to be EM major. At day 16, the steroid pulse therapy with methylprednisolone (1000 mg/day) was conducted for three successive days from day 19 to day 21, followed by prednisolone (30 mg/day) (Fig. 3). However, the symptoms were still not improved and prednisolone was increased to 50 mg/day (1 mg/kg body weight) (Fig. 3). Then, her skin lesions were gradually improved, and the dosage of prednisolone was tapered off from day 29 (Fig. 2F, G). The skin care provided by dedicated nursing during this period contributed to this improvement as shown in the ASCO guideline [6]. Photographing the lesions over time also helped control the disease.

In May 2023, CT evaluation showed disease progression in the primary tumor, intrapelvic masses, skin metastases, and bone metastases. Pem was discontinued due to its inefficacy and severe skin toxicity. After recovery from the skin toxicity, the patient was transferred to a palliative care hospital in June 2023.

## Discussion

According to UpToDate^®^, EM is defined as an acute, immune-mediated condition characterized by the appearance of distinctive target-like lesions on the skin. These lesions are often accompanied by erosions or bullae involving the oral, genital, and/or ocular mucosae. EM major is an EM with severe mucosal involvement and may have associated systemic symptoms, such as fever and arthralgias [7]. Considering the clinical and pathological presentation described above, the present case is compatible with EM major. A complete list of the patient’s medications was reviewed, and the other drug-induced causes were ruled out. Skin toxicity developed as an irAE is classified into the following three categories according to the ASCO guidelines [6]: RID, BD, and severe cutaneous adverse reactions (SCAR) [6]. EM is included in RID. EM is a targeted reaction of the skin and mucous membranes usually caused by infections, such as HSV, but it can also be associated with immune-related drug eruptions.. In the present case, some lesions were destructed, and they were easily infected. There was no history or suspicion of autoimmune diseases, such as systemic lupus erythematosus and dermatomyositis, or allergic reaction.

In this case, the BP180 level was not elevated. BP180 is usually elevated in bullous pemphigoid. The sensitivity and specificity of BP180 are 84.4% and 98.9%, respectively, in diagnosing bullous pemphigoid [8]. However, Sadik et al. reported that the positivity of BP180 was observed in four out of nine bullous pemphigoid cases, which presented as skin irAE, from six German dermatology centers [9]. In the present case, BP180 could not be used as a marker of BD induced with Pem. Clinical monitoring using serial photographs of the skin lesion was helpful in the present case.

Some skin irAEs exhibit very diverse presentations, and in some cases, as in the present case, it seems difficult to differentiate between RID and BD. A skin biopsy is a very important source of information as recommended by the ASCO guidelines [6].

**Fig. 4:**
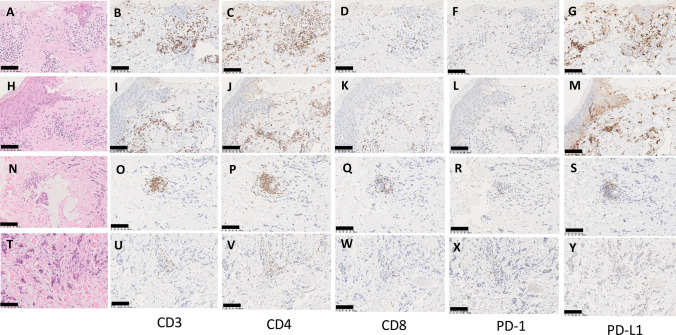
Immunohistochemical staining of the skin lesions. Two “Bullous Dermatoses” lesions are indicated in (**A**–**M**). Skin metastasis lesions are indicated in (**N**–**R**). Cancer infiltration lesions in to skin are indicated in (**T**–**Y**). Hematoxylin and eosin (HE) staining are (**A**, **H**, **N**, **T**). Immunohistochemistry (IHC) with anti-CD3 antibody is (**B**, **I**, **O**, **U**). IHC with anti-CD4 antibody is (**C**, **J**, **P**, **V**). IHC with anti-CD8 antibody is (**D**, **K**, **Q**, **W**). IHC with anti- PD-1 antibody is (**F**, **L**, **R**, **X**). IHC with anti- PD-L1 antibody is (**G**, **M**, **S**, **Y**). The black bar indicates 100 μm

The Grade 3 BD is more clearly defined as “skin sloughing covering > 30% BSA” in the guideline [6]. If there is the pathologically confirmed lesion of bullous pemphigoid, it seems better to start the treatment for bullous pemphigoid as a management of BD. Immunostaining of the bullous lesion of the present case was performed with anti-CD3 (Roche, Clone 2GV6, ready to use), CD4 (Roche, Clone SP35, ready to use), and CD8 (Clone C8/144B, ready to use) antibodies and anti-PD (Roche, Clone NAT105, ready to use) and PD-L1 (Roche, Clone 28–8, ready to use) antibodies. Strong infiltration of CD3 + , CD4 + , and CD8 + T cells was observed in this lesion, and the PD-L1 expression in keratinocytes was also increased (Fig. 4A–M). For comparison, we performed immunostaining of non-bullous lesions, but the infiltration of CD3 + , CD4 + , and CD8 + T cells was localized around the vessels, and there was also no increased PD-L1 expression in the keratinocytes (Fig. 4N–Y). Abundant PD-L1 expression in keratinocyte and CD4 + lymphocyte infiltration were observed in bullous lesions (Figs. 4C, J, G, M and supplemental figure).

EM major and SCAR are completely different diseases to each other, however the early clinical presentations of SJS and TEN are similar to that of EM major [10]. Pem rarely causes EM (4%) [4]. EM major has been rarely reported with sequential use of nivolumab and ipilimumab [11]. In this case report, the EM major was not controlled with the standard dosage of steroids and was treated with immunoglobulins and higher doses of steroids. Our case also followed a similar course to this case, except for the use of immunoglobulins. There are no reports that clearly distinguish between EM major and SCAR based on differences in the infiltration of immunocompetent lymphocytes and the expression of immune checkpoint molecules. The only exception was described regarding the extent of CD4 + lymphocyte infiltration in EM major and SJS/TEN, with EM major having a relatively higher infiltration of CD4 + lymphocytes compared to SJS/TEN (p = 0.0017) [10]. There is no literature that clearly describes how the infiltration of CD4 + lymphocytes causes EM major. In the above-mentioned case report, the involvement of CD4 + , CD8 + and Foxp3 + lymphocytes were indicated, but IHC data were not available. Recently, it has been shown that CD4 + lymphocytes play an important role in the mechanism of ICI-induced destructive thyroiditis [12]. According to this study, CD4 + lymphocytes are braked through PD-1/PD-L1 interaction with dendritic cells, B cells, natural killer cells, macrophages, and thyroid follicular cells. Once anti-PD-1/PD-L1 antibodies release this brake, CD4 + lymphocytes secrete granzyme via T cell receptor -antigen-HLA class II complex, exerting a cell-killing effect of thyroid follicular cells. We imagine that a similar reaction occurs to keratinocytes in the case of EM major. In our case, keratinocytes in EM major lesions highly express PD-L1, suppressing the cell-killing effect of CD4 + lymphocytes; we would like to consider the possibility that a strong cellular effect was exerted by Pem. The existence of sensitized keratinocytes appears to be necessary prior to the origin of EM major, and we cannot deny the possibility that some preceding event induced it. For example, in this case, irradiation of the skin metastases may have played a role. The relationship between irradiation and ICI-induced EM major is unclear, although the case report does exist [13]. EBI was performed on the skin metastasis three times before the onset of irAE in the present case. Twenty-one reports have shown that irradiation causes skin irAEs [14].

Why did severe skin irAEs occur in the present case? The other possible triggering factors are as follows. This case had very widespread skin metastases, which may be triggered by skin irAEs. However, we cannot find these facts in the literature. Further, self-destruction of the skin metastatic lesions and the subsequent skin infection may trigger the development of skin irAEs. However, it was reported that the associations with viral and bacterial infections are considered rare and deceptive [15].

In conclusion, PD-L1 expression in keratinocyte and infiltration of CD4 + lymphocyte can predict a severe type of erythema multiforme major induced by Pem (Table 1).

**Table 1:** The presence of CD4^+^ T cells and PD-L1^+^ keratinocytes in the skin biopsy, markers of Erythema Multiforme major (EM major)

Cases	Findings	Primary, ICI	Reference
10 patients with SJS, 4 patients with 10 vs 16 patients with EM major	The number of CD4 + cells⁄5 high-power fields in SJS⁄TEN was significantly lower than in EM Major (P = 0.0017)	Unknown	Ref. 10
37 year, Female	CD8 + , CD4 + and Foxp3 + lymphocyte infiltration	melanoma, ipilimumab and nivolumab	Ref. 11
Our case	Female, 70 year	Anal Canal Ca, Pem	Kado

*SJS* Stevens-Johnson syndrome, *TEN* toxic epidermal necrolysis, *Pem* pembrolizumab

### Supplementary Information

Below is the link to the electronic supplementary material.

Supplementary file1 (PDF 3057 KB)

## Data Availability

All data can be available upon request.

## References

[1] Keung EZ, Wargo JA (2019). The current landscape of immune checkpoint inhibition for solid malignancies. Surg Oncol Clin N Am.

[2] Marcus L, Fashoyin-Aje LA, Donoghue M (2021). FDA approval summary: pembrolizumab for the treatment of tumor mutational burden-high solid tumors. Clin Cancer Res.

[3] Yin Q, Wu L, Han L (2023). Immune-related adverse events of immune checkpoint inhibitors: a review. Front Immunol.

[4] Dos Santos M, Garrett NF, Carvalho da Costa AC, Barros Ferreira E (2021). Prevalence of dermatological toxicities in patients with melanoma undergoing immunotherapy: systematic review and meta-analysis. PLoS One.

[5] Jamora MJJ, Jiao D, Bystryn JC (2003). Antibodies to desmoglein 1 and 3, and the clinical phenotype of pemphigus vulgaris. J Am Acad Dermatol.

[6] Schneider BJ, Naidoo J, Santomasso BD (2021). Management of immune-related adverse events in patients treated with immune checkpoint inhibitor therapy: ASCO guideline update. J Clin Oncol.

[7] https://www.uptodate.com/contents/erythema-multiforme-pathogenesis-clinical-features-and diagnosis?search=Erythema%20multiforme&source=search_result&selected Title=1~150&usage_type=default&display_rank=1

[8] Mai Y, Izumi K, Mai S, Ujiie H (2022). The significance of preclinical anti-BP180 autoantibodies. Front Immunol.

[9] Sadik CD, Langan EA, Gutzmer R (2020). Retrospective analysis of checkpoint inhibitor therapy-associated cases of bullous pemphigoid from six german dermatology centers. Front Immunol.

[10] Iwai S, Sueki H, Watanabe H (2012). Distinguishing between erythema multiforme major and Stevens-Johnson syndrome/toxic epidermal necrolysis immunopathologically. J Dermatol.

[11] Utsunomiya A, Oyama N, Iino S (2018). A case of erythema multiforme major developed after sequential use of two immune checkpoint inhibitors, nivolumab and ipilimumab, for advanced melanoma: possible implication of synergistic and/or complementary immunomodulatory effects. Case Rep Dermatol.

[12] Yasuda Y, Iwama S, Sugiyama D (2021). CD4+ T cells are essential for the development of destructive thyroiditis induced by anti-PD-1 antibody in thyroglobulin-immunized mice. Sci Transl Med.

[13] Ambur AB, Mammino J, Nathoo R (2021). Recurrent erythema multiforme induced by the combination of pembrolizumab and radiation. Cureus.

[14] Kaszycki MA, Leventhal J (2021). Review of immune checkpoint inhibitors and radiotherapy related skin toxicities. J Dermatol Skin Sci.

[15] Gudiol C, Hicklen RS, Okhyusen PC (2022). Infections simulating immune checkpoint inhibitor toxicities: uncommon and deceptive. Open Forum Infect Dis.

