# Supplementary figures and images for: PD-L1 expression in keratinocyte and infiltration of CD4 + T lymphocyte can predict a severe type of erythema multiforme major induced by the anti-PD-1 antibody, pembrolizumab
Source: Int Cancer Conf J. 2024 Mar 29;13(3):268–74. doi: 10.1007/s13691-024-00676-4 (PMC11217243; doi:10.1007/s13691-024-00676-4)

## Supplemental Figure

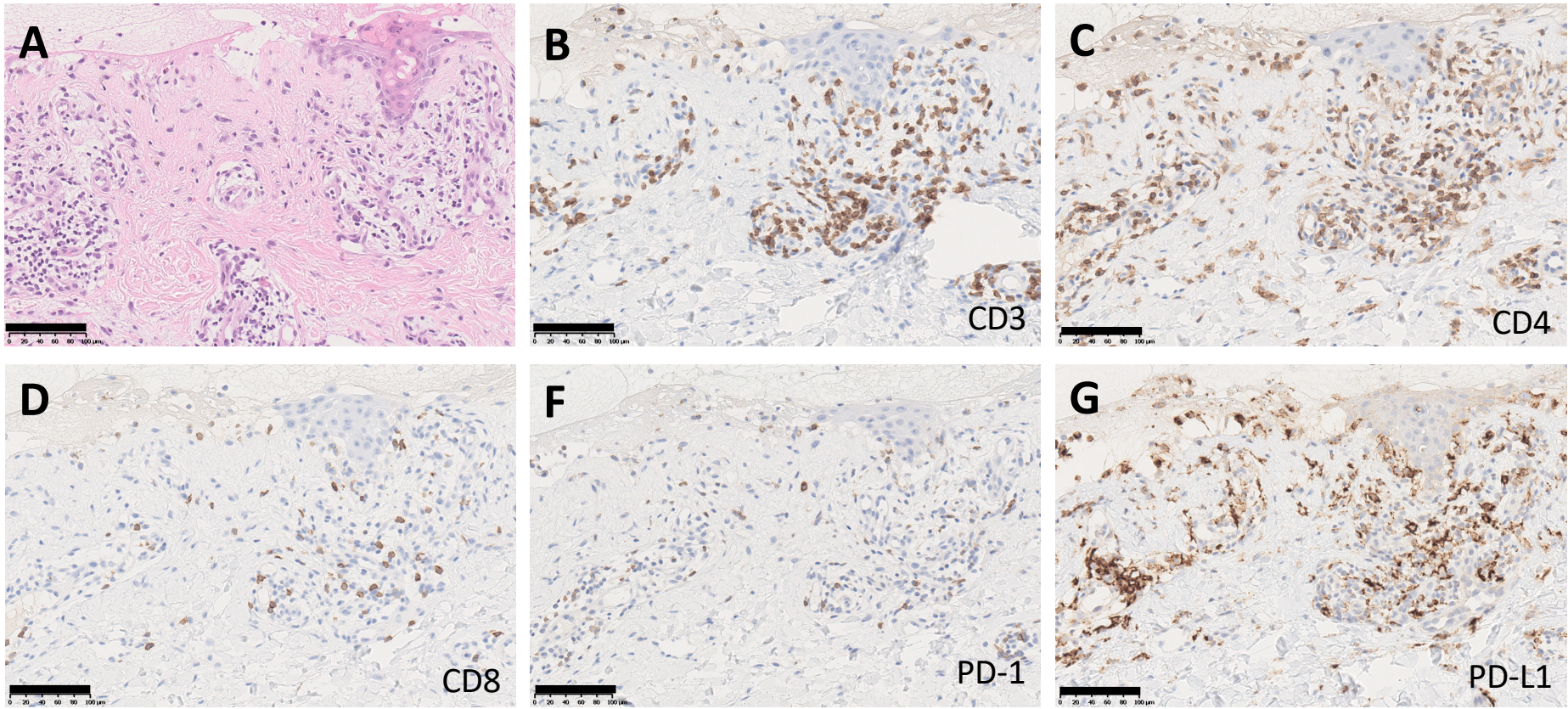

Supplement: Supplementary file 1 — Supplementary file1 (PDF 3057 KB) [file 13691_2024_676_MOESM1_ESM.pdf]
